# Sympathy

**Published:** 1887-01-22

**Authors:** 


### Words of Consolation.

[Communications for this column should be addressed, "Chaplain," care of the Editor of The Hospital, who will gratefully welcome any suggestions or inquiries from matrons, nurses, and the staff generally.]

XVII.—Sympathy in the Physician.

The Spirit of Christ is the fountain of sympathy. The best Physician the world has ever known was, and is, perfect in sympathy. He, therefore, who would excel as a physician must be a follower of Christ, must look upon his calling as very sacred, must believe that his suffering patients have souls as well as bodies. One would have thought that the more carefully a man studied the vast problems of flesh and blood, the further he explored the depths of medical science, or strove to grapple with the mysterious forces connected with nerve centres and the brain, the less satisfied he would be with bare materialistic theories. But, alas! it is not always so. We have reason to fear that some few of our leading physicians base their science almost exclusively upon matter, making little allowance for mind, none at all for spirit. We cannot argue with them in this journal. We prefer to address ourselves to a much larger number whom we credit with decided leanings to Christianity, but who might become far more powerful and nobler men in their profession (in the best sense) by exercising faith in Christ.

The offices of the Physician and the Clergyman are of course distinct. But if we hold that human nature is spiritual as well as material, we shall find the two ministries crossing one another in the same person, or, better still, blended, as in our Lord's ministry. What would become of many of our poor, especially in distant and scattered rural districts, were not the clergy, as a rule, ready at any moment to do their best in cases of emergency, accident, or sudden illness? While in hundreds of parishes the people are only too glad, where nothing much is the matter, yet enough to create a demand for medicine, to make a dispensary of the Clergyman's house. Some of the medicines dispensed in this way may be needless; some are harmless; some do good; some are patent medicines, given with little or no discretion, and, therefore, do harm. But this fact, together with the amount of nourishment the sick poor receive in the shape of food and wine, shows how thoroughly ministrations to body and soul are blended in the lives of our clergy. We should hardly expect a similar correspondence in our physicians. But we venture to maintain that something of the kind is highly desirable. The clergyman or chaplain even in Hospital is not always the first to know of a case needing religious offices. Many medical men, thorough Christians at heart, have borne witness to the calls made upon them by distressed souls when no clergyman was at hand, and told how they have done their best with Bible, or Prayer Book, or without either, to respond to such calls. It is never too late for a physician to begin to think of his high calling being turned to some spiritual account, as the following anecdote by a surgeon in the American army will show.

I never felt the need of being a Christian so much as at the battle of ———. A number of men were brought into a tent where we were amputating limbs, and probing wounds. Examining the hurts of one poor fellow, I was obliged to tell him he could live but a few minutes. He turned and looked to me: "Surgeon, are you a Christian?" I had to confess I was not. "Is there no Christian here?" No one responded. "I want some Christian to pray with me before I die."—"Are you a Christian?" I inquired. "Oh, yes, sir! I am a Christian: but I should so love to have some one to pray with me before I go. O surgeon! won't *you* pray?" The pleading of the dying man was more than I could resist. I knelt down beside him, and offered up a heartfelt prayer to God. I don't know much about such things; but that prayer has had a most marked influence on my life ever since. The soldier died in a few minutes after its close.

(*To be continued.*)

